# Tools and criteria to select patients with advanced Parkinson’s disease for device-aided therapies: a narrative review

**DOI:** 10.1007/s00702-023-02656-z

**Published:** 2023-07-27

**Authors:** Harmen R. Moes, Tove Henriksen, Jarosław Sławek, Onanong Phokaewvarangkul, Erik Buskens, Teus van Laar

**Affiliations:** 1 Department of Neurology, University of Groningen, University Medical Center Groningen, 9713 GZ Groningen, The Netherlands; 2 Department of Neurology, Movement Disorder Clinic, Bispebjerg Hospital, Copenhagen, Denmark; 3 Department of Neurology, St Adalbert Hospital Copernicus, Gdansk, Poland; 4 Department of Neurological and Psychiatric Nursing, Faculty of Health Sciences, Medical University of Gdansk, Gdansk, Poland; 5 Chulalongkorn Center of Excellence for Parkinson Disease & Related Disorders, Department of Medicine, Faculty of Medicine, Chulalongkorn University and King Chulalongkorn Memorial Hospital, Thai Red Cross Society, Bangkok, Thailand; 6 Department of Epidemiology, University of Groningen, University Medical Center Groningen, Groningen, The Netherlands

**Keywords:** Parkinson’s disease, Device-aided therapies, Screening tools, DBS, LCIG, CSAI

## Abstract

This article provides an overview of the various screening and selection tools which have been developed over the past 25 years to identify patients with Parkinson’s disease (PD) possibly eligible for device-aided therapies (DATs). For the available screening tools, we describe the target therapies (subtypes of DAT), development methods, validation data, and their use in clinical practice. In addition, the historical background and potential utility of these screening tools are discussed. The challenges in developing and validating these tools are also addressed, taking into account the differences in population, the local health care organization, and resource availability.

## Introduction

Parkinson’s disease (PD) is a fast-growing neurological disorder with an estimated global prevalence of 12.9–14.2 million patients in 2040 (Dorsey and Bloem 2018). To date, there is no cure for PD (Armstrong and Okun 2020). During the first years after being diagnosed with PD, patients benefit from oral treatments, including various forms of dopaminergic medication, with levodopa being the first-choice treatment (de Bie et al. 2020).

Over time, narrowing of the therapeutic window leads to motor complications such as wearing-off and dyskinesias. These predictable fluctuations are treated by extra doses of levodopa, or by adding a dopamine agonist, MAO-B inhibitor, or COMT inhibitor, while dyskinesias can be minimized by reducing the dose of dopaminergic medications, or adding amantadine (Deuschl et al. 2022; Fabbri et al. 2023).

However, many patients develop unpredictable fluctuations and/or troublesome hyperkinesia over the course of their disease which cannot be treated by just changing the oral or transdermal medication. At that stage of disease, a patient may be eligible for a device-aided therapy (DAT) (Deuschl et al. 2022). Currently, there are four different DATs available in the Western world: (1) deep brain stimulation (DBS), (2) continuous subcutaneous apomorphine infusion (CSAI), (3) levodopa-carbidopa intestinal gel infusion (LCIG) and (4) levodopa-carbidopa-entacapone intestinal gel infusion (LECIG) (Nyholm and Jost 2022; Fabbri et al. 2023). The shared mechanism of action of CSAI, LCIG, and LECIG is based on the presumed mechanism of effect of ‘continuous dopaminergic stimulation’ (Wolters et al. 2008; Antonini and Tolosa 2009; Senek and Nyholm 2014). The DAT armamentarium will be expanded in the near future with continuous subcutaneous administration of foslevodopa-foscarbidopa (Soileau et al. 2022; Fabbri et al. 2023).

At this point, it should be noted that progression of PD towards the phase of so-called advanced PD is also associated with the occurrence of motor and non-motor symptoms that are not responsive to levodopa (Coelho and Ferreira 2012). These dopa-resistant symptoms include non-motor symptoms, such as dementia or dysautonomia, and axial motor symptoms, such as postural instability and falling. DATs are primarily effective for dopa-responsive motor complications, with adjunctive beneficial effects on some non-motor symptoms (Marsili et al. 2021a; Deuschl et al. 2022). Consequently, DATs are not effective in treating dopa-resistant symptoms (Odin et al. 2015).

To date, the different DATs have never been compared with each other in a clinical randomized head-to-head study (Marsili et al. 2021a). However, two recently published comprehensive network meta-analyses did show that STN-DBS is the most effective DAT, although the results of both analyses do not completely overlap (Antonini et al. 2022; Rajan et al. 2022). In addition, the results of a randomized trial comparing DBS and LCIG for cost-effectiveness are likely to be published in the near future (Van Poppelen et al. 2020). The most optimal patient profiles for the separate DATs have been developed based on efficacy and safety data from randomized controlled trials with the separate DATs. For example, DBS is not suitable for patients with cognitive impairment, but in contrast to the other DATs, DBS offers remarkable therapeutic benefits for PD patients with therapy-resistant tremor (Antonini et al. 2018; Deuschl et al. 2022).

Since the introduction of DBS and apomorphine in the 1990s, several treatment algorithms for neurologists have been developed, providing guidance on which DAT would be most suitable for which patient, including the presence of various motor and non-motor symptoms (Pollak 2013; Worth 2013; Erasmi et al. 2014; Odin et al. 2015; Dietrichs and Odin 2017; Williams et al. 2017; Fabbri et al. 2018; Antonini et al. 2018). In addition to clinical characteristics, patient preferences and expectations are also paramount in choosing a specific DAT (Nijhuis et al. 2016, 2019; Geraedts et al. 2019).

Moreover, since 2000 screening criteria were developed in order to refer patients in a timely manner to centers of expertise (Okun et al. 2004; Moro et al. 2009; Worth 2013; Luquin et al. 2017; Antonini et al. 2018). These criteria are important, especially for general neurologists also treating PD patients, to identify patients as possible candidates for DAT (Siddiqui et al. 2018). Some of the screening criteria were developed specifically to identify possible candidates for DBS (e.g., FLASQ-PD and Stimulus), while others assess possible eligibility for any DAT (e.g., CDEPA, 5-2-1 criteria, MANAGE-PD) (Okun et al. 2004; Moro et al. 2009; Luquin et al. 2017; Santos-Garciá et al. 2020; Antonini et al. 2021). The impetus for creating these types of screening criteria came in part from the industry behind DATs. For example, the development and marketing of Stimulus have been funded by Medtronic (Moro et al. 2016), while MANAGE-PD is a project supported by AbbVie (Antonini et al. 2021).

The development process of a screening tool is determined by the purpose for which it is intended. The measures of diagnostic accuracy—sensitivity, specificity, positive predictive value and negative predictive value—are important in this context (Lewis et al. 2015). The trade-off between high sensitivity and high specificity is relevant. If the aim is to ensure that all patients eligible for DAT are identified by non-experts, a high sensitivity is preferred, which may lead to a high rate of inappropriate referrals and consequently false expectations among patients. If the priority is to minimize the number of inappropriate referrals, high specificity and an adequate positive predictive value are preferred, with under-referral and missed opportunities as possible drawbacks. Therefore, a balance must be struck between these two extremes.

Considering the above, the question arises as to which of the available screening tools is most useful in clinical practice to appropriately refer patients for any DAT. This review will provide an overview of the currently available screening methods and will compare the methodologies used to develop these tools, their validity and their overall value in clinical practice. We will not discuss the effectiveness or the optimal patient profiles for the various DAT subtypes, as this has already been described elsewhere by others (Siddiqui et al. 2018; Antonini et al. 2018; Deuschl et al. 2022).

## Methods

Relevant articles for all available time periods were retrieved from the MEDLINE database using PubMed^®^ on January 21, 2023. We used the following query: “(Parkinson’s disease) AND [DBS OR apomorphine OR intestinal gel OR (device-aided therapy)] AND [referral OR candidates OR eligible OR (patient selection) OR identification OR 5-2-1] NOT dystonia NOT essential tremor NOT Tourette OR [(5-2-1 OR CDEPA) AND advanced Parkinson’s]”. We exported the retrieved articles to Mendeley Desktop (version 1.19.8).

The primary search yielded 595 articles. The selected articles were screened for relevance by HM, based on title and abstract, and mainly looking for articles that formulated pre-selection/screening criteria, preferably including diagnostic accuracy measures.

Based on title and abstract, 462 articles were excluded. The contents of the remaining 133 articles were analyzed for relevant data. We selected possible screening methods and presented them in a table. No meta-analysis was performed. Methods capable of distinguishing between patient categories within PD, but not specifically developed for identifying possible candidates for DAT, were briefly discussed separately.

If cross-referencing yielded additional articles that had not yet been included, they were added (*n* = 14). In addition, nonsystematic searches were conducted using Google Scholar to find articles that referenced articles we had already included (*n* = 8).

## Usefulness of screening tools

Screening PD patients for their eligibility for DAT is focused on timely identification for referral to an expert center (Odin et al. 2015; Moro et al. 2016). It is important to realize that “eligibility for DAT” is not the same as “eligibility for referral to a specialized center for further assessment” (Moro et al. 2016). Eligibility assessment in specialized centers, regardless of the type of DAT, often places a physical and emotional burden on the patients and their families. User-friendly referral criteria may reduce the number of inappropriate referrals, while also avoiding under-referral of potentially suitable candidates (Moro et al. 2016; Antonini et al. 2018; Deuschl et al. 2022; Fabbri et al. 2023).

The importance and potential usefulness of unambiguous and user-friendly screening tools is affirmed by several partially overlapping observations, like (1) the existence of practice variation, (2) undertreatment, (3) lack of expertise among neurologists, sometimes leading to over-referral, (4) unawareness of patients about DAT, and finally (5) the limited use of available tools. We will discuss each observation, referring to relevant data from the literature.

### Practice variation

The availability and frequency of DAT application varies greatly between countries (Ezat et al. 2017; Henriksen et al. 2020; Crispo et al. 2020; Norlin et al. 2021). Possible explanations include differences in access to care, specific referral pathways, health-seeking behavior, or the need for DAT (Crispo et al. 2020). Obviously, resource availability determines the use of DAT and may thus be a factor in practice variation. However, inadequate identification of eligible patients is another factor. This is supported by the international observational OBSERVE-PD study, which showed a large variation between countries in the proportion of patients identified as having advanced PD (Fasano et al. 2022). This variation in case finding may be reduced by establishing clear screening criteria.

### Under-referral

Analysis of data from the Romanian OBSERVE-PD cohort suggests that 54.3% of patients eligible for DAT do not actually receive DAT (Szasz et al. 2021). In Poland infusion therapies are available since 2018, but after 5 years less than 300 patients have been treated, due to a lack of knowledge on identification and definition of advanced PD (i.e. more than 50% of the day with off-periods and/or disabling dyskinesias) (Data from the National Health Fund of Poland (NFZ). Also in the Middle East, North Africa and South Asia, there is a need for more DAT, in addition to the need for more movement disorders specialists (Khalil et al. 2020).

Importantly, some studies have revealed evidence of a specific underrepresentation of women in the overall group of patients referred for DBS (Jost et al. 2022). It was pointed out that gender ratios in DBS cohorts better reflect the gender ratios in the overall PD population when patients receive specially developed educational materials and referring physicians use DBS screening tools (Jost et al. 2022).

### Lack of expertise among general neurologists

Several studies show evidence of a lack of expertise among general neurologists regarding the indications for DAT. This is particularly evident in studies of neurologists’ views on DBS. For example, a 2017 German survey found that only 41% of neurologists knew the specific criteria for considering DBS as a treatment option (Lange et al. 2017). Another study among neurologists in the northeastern part of the Netherlands found that only 47% of these neurologists considered themselves sufficiently skilled to determine eligibility for DAT in PD patients (Moes, unpublished findings). Similarly, a U.S. survey in 2021 showed that movement disorder specialists and general neurologists had different views on the minimum disease duration and required medication adjustments prior to agreeing on an indication for DBS (Cabrera et al. 2021).

Lack of knowledge may be the cause of under-referral, but it may also lead to inappropriate referrals. Although there are no systematic reviews on the percentage of inappropriate referrals for DAT, there are some figures on referrals for DBS. Three studies from different countries [Germany and Spain (2011); the Netherlands (2019); Russia (2021)] show that among PD patients referred for DBS, the percentage of patients who are rejected ranges from 26 to 79%, with the lowest percentage in the Netherlands and the highest in Russia (Wächter et al. 2011; Geraedts et al. 2019; Bril et al. 2021). Meanwhile, the first reports have appeared that claim that the use of a screening tool may reduce the number of inappropriate referrals (Wächter et al. 2011).

The apparent lack of specific knowledge among neurologists is a growing concern considering the increasing number of patients with PD worldwide. An international survey including 44 movement disorders specialist from around the world found that the 75% of respondents believed that in the coming years the number of patients treated with DAT will increase, but 57% of them indicated there is a lack of proper guidelines to identify suitable PD patients for DAT. Interestingly, movement disorders specialists from North America were more positive about proper DAT guidelines than their European colleagues (Marsili et al. 2021b).

The data on the FLASQ-PD demonstrated that screening tools contribute to appropriate referral (Oyama et al. 2012). This screening tool was better at identifying PD patients eligible for DBS than the clinical impression of general neurologists (Oyama et al. 2012).

### Unawareness of patients about DAT

A large 2011 survey involving 3327 Swedish PD patients found that 80% of those with advanced PD had heard of the possibility of DAT, but only 27% had received information about DAT from their physician (Lökk 2011). A survey in Dutch PD patients (121 respondents) who had started treatment with DAT during the previous 3 years, showed that 59% of respondents had not been informed about alternative treatment options (Nijhuis et al. 2019).

### Limited use of available tools

Irrespective the presence of DAT-related screening tools, such as FLASQ-PD, Stimulus, and MANAGE-PD, it was shown by a recent survey in the USA that these tools are not widespread and do not assist in a proper timing of at least DBS (Cabrera et al. 2021). Possibly this statement is also true for other DATs.

## Screening criteria and screening tools

### Evaluation of available tools

Below, we discuss the available screening tools and screening criteria for identifying PD patients who are possible candidates for DAT. We have divided the different tools into three categories, namely screening tools for advanced PD (the CDEPA-questionnaire and the 5-2-1 criteria), screening tools for eligibility for DAT-referral in general (MANAGE-PD and D-DATS), and tools for identifying potentially eligible candidates for DBS (FLASQ-PD and Stimulus). Table 1 summarizes the characteristics of the different tools and criteria. In “Head-to-head comparison”, we discuss available head-to-head comparisons of different screening tools. Finally, in “Other screening tools and testing methods”, we briefly discuss other PD selection methods that we believe are inadequate as screening tools for general neurologists in assessing potential DAT eligibility (Defer et al. 1999; Heldman et al. 2016; García et al. 2022; Barer et al. 2022).

#### Screening tools for advanced PD

##### CDEPA-questionnaire

In 2017 the results of a Spanish initiative to establish a definition of advanced PD were published (Luquin et al. 2017). The authors noted that “it is of interest to know the patients’ clinical characteristics defining advanced PD, making them eligible for DAT.” The results of the study resulted in the CDEPA questionnaire (CDEPA is an abbreviation for the Spanish description “Cuestionario De Enfermedad de Parkinson Avanzada” [Questionnaire for Advanced Parkinson’s Disease].

The CDEPA is based on a 3-round Delphi Study during 2013–2014 and involved 240 Spanish neurologists and 26 Spanish experts in movement disorders. Views on the clinical features of advanced PD were collected in several rounds, with data collection based on a 33 questions. The researchers defined possible symptoms signifying advanced PD. The symptoms of the CDEPA are presented in a matrix arranged on the horizontal axis into three probability categories (definite symptoms, probable symptoms, possible symptoms), and on the vertical axis grouped into 6 domains ([1] general characteristics of the disease, [2] disability, [3] motor symptoms related to the treatment, [4] motor symptoms related to the disease, [5] non-motor symptoms related to the disease, and [6] neuropsychiatric and cognitive manifestations.

Advanced PD is diagnosed when a patient has at least one definite symptom, or at least two probable symptoms. Combining one possible symptom from the “motor or nonmotor symptoms related with the disease” domain with one possible symptom from the “neuropsychiatric and cognitive” domain is equivalent to one probable symptom.

The authors state that advanced PD is “an advanced stage of PD in which certain symptoms and complications are present, with a detrimental influence on the overall patient’s health conditions and with a poor response to conventional treatments” (Luquin et al. 2017). Because of the level of response to conventional treatments, eligibility for DAT implicitly follows from the proposed definition.

The CDEPA questionnaire was validated in a prospective cohort of 173 patients with PD (Martinez-Martin et al. 2018). They had a disease duration of at least 2 years and were not yet treated with DAT. The study was conducted in 24 Spanish hospitals. The reference test (gold standard) in this validation study was determined by one neurologist from the participating hospital, while the CDEPA questionnaire was administered by a fellow neurologist, who was blinded to the judgment of the reference test. The prevalence of advanced PD in the study cohort was 37.6%. In this validation study, the CDEPA questionnaire had a sensitivity of 96.9% for correctly identifying advanced PD. The specificity of the CDEPA was 57.4% with a positive predictive value of 57.8%. However, the high prevalence of advanced PD in this study means that the reported sensitivity and specificity may not be realistic in clinical populations with a lower prevalence (Leeflang et al. 2009).

The CDEPA was used to examine the prevalence of advanced PD in Spain in a cohort of 929 PD patients, in 21 different hospitals (both general hospitals and third-line centers) (Martínez-Castrillo et al. 2021). The CDEPA was used to determine whether the patient had advanced PD, but the neurologist could overrule this judgment (judgment of the neurologist was the gold standard). The prevalence of advanced PD in Spain was estimated at 38.2% in this study.

In total 355 patients were classified as having advanced PD, whereas 54 patients (15.2%) were treated with DAT. Characteristics which predicted the suitability for DATs were disease duration > 10 years, OFF symptoms > 25% of the day with ADL restriction, and older age (Martínez-Castrillo et al. 2021). The 301 patients with advanced PD who were not treated with DAT were clinically stable in 33.2%, in 26.6% the option of DAT was not considered by the clinician, 12.6% had contraindications for DAT, 13.3% had rejected DAT, and 7.6% were on a waiting list for DAT. In 6.6% of patients with advanced PD, there was another reason they were not treated with DAT.

##### The 5-2-1 criteria

The 5-2-1 criteria serve as a rule of thumb to determine whether a patient has advanced PD. The rule refers to ≥ 5 doses of oral levodopa per day and/or ≥ 2 h of “off” time per day, and/or ≥ 1 h of troublesome dyskinesia per day. The first scientific publications on the 5-2-1 criteria appeared in 2020 (Santos-Garciá et al. 2020; Aldred et al. 2020), whereas Antonini introduced the term ‘5-2-1 criteria’ already in 2017, at a Swedish congress (Nordlund 2018).

The 5-2-1 criteria are based on a consensus statement by European PD experts who developed criteria for advanced PD and for eligibility for DAT, based on a Delphi panel (Antonini et al. 2018).

The Delphi consensus document lists 15 clinical indicators of patients with possible advanced PD and 7 characteristics of patients with advanced PD, making them suitable for DAT (Antonini et al. 2018). The components of the 5-2-1 criteria are three of the six clinical motor indicators for advanced PD.

No explanation is provided in the Delphi consensus document why the 5-2-1 criteria consists of precisely those specific three components and does not include for instance the criterion ‘moderate level of troublesome dyskinesia’ (Antonini et al. 2018).

The components of the 5-2-1 criteria were also mentioned in a survey of 103 international experts, being part of the NAVIGATE-PD program, suggesting possible criteria to refer patients for DAT (Odin et al. 2015). However, their criteria also included the requirement for medication optimization. Some patients with marked ‘off’ symptoms should be considered for referral even if their overall ‘off’ duration appears acceptable. If symptoms continue to be refractory or intolerable side-effects develop, and motor fluctuations accompanied by troublesome dyskinesias persist despite amantadine (100–400 mg/day, if available), referral for DAT should be considered, including patients with a disease duration < 4 years (Odin et al. 2015).

The appealing aspect of the 5-2-1 criteria is that this rule of thumb is easy to remember and therefore seemingly easy to apply in clinical practice. However, the link between the 5-2-1 criteria and advanced PD is explained in different ways. Some believe that the rule is a disjunction, while others interpret it as a conjunction. A disjunction means that the three components (5, 2, 1) are paired with the Boolean operator ‘OR’, which makes the collection large and inclusive. Conversely, a conjunction means that the components are paired with the Boolean operator ‘AND’, which leads to a small, specific collection. An example of the former interpretation is the validation study from 2022 (Malaty et al. 2022), while the conjunction interpretation is applied by Hauser et al. (Hauser et al. 2022).

The difference between interpretation as disjunction or conjunction is evident from several studies. The Japanese JAQPAD study (2021), which examined the quality of life of patients with advanced PD, included 1599 patients who met at least one of the 5-2-1 criteria. In total 158 patients (9.9%) met all three criteria (Hayashi et al. 2021). An international study of advanced PD patients treated with LCIG reported that 98% of the study group (*N* = 82) met at least one of the 5-2-1 criteria, while only 20% met all three 5-2-1 criteria (Aldred et al. 2020).

A validation study of the 5-2-1 criteria was published in 2022 (Malaty et al. 2022). Diagnostic accuracy for diagnosing advanced PD was examined in a population of 4714 patients from 7 countries. The 5-2-1 criteria were compared with the judgment of treating neurologists. A patient had advanced PD if he/she had at least one of the 5-2-1 criteria (disjunction). Overall 33% of patients (*n* = 1546) met one of these 5-2-1 screening criteria for advanced PD. However, the treating neurologists classified only 702 patients as having advanced PD (14.9%), resulting in a positive predictive value value of 35.7% (Malaty et al. 2022).

#### Screening tools for eligibility for DAT-referral

##### MANAGE-PD

In 2021, the MANAGE-PD tool was published (Antonini et al. 2021). MANAGE-PD is an acronym for Making Informed Decisions to Aid Timely Management of Parkinson’s Disease. This is a clinician-based tool designed to facilitate timely identification and treatment of patients with advanced PD with suboptimal symptom control during standard therapy. The tool can be accessed at http://www.managepd.com and at http://www.managepd.eu.

The MANAGE-PD was developed for general neurologists to determine whether treatment of PD should be optimized and whether the patient is possibly eligible for DAT (Antonini et al. 2021). The outcome of the tool classifies patients into three possible categories. Category 1: the patient's symptoms are adequately controlled with the current therapy; category 2: PD symptoms are inadequately controlled with current oral therapy and optimization of oral therapy is recommended; category 3: PD symptoms are inadequately controlled with current oral therapy and in addition to optimization of oral therapy, evaluation for DAT is recommended.

The MANAGE-PD is an online tool that requires the user to answer several questions in two different sections. The first section screens whether the patient has adequate control of symptoms with current oral therapy. This section consists of five questions: (1) does the patient use levodopa ≥ 5 times per day?; (2) does the patient have ≥ 2 h of "off" symptoms per day?; (3) does the patient have unpredictable fluctuations of motor symptoms?; (4) does the patient have bothersome dyskinesias?; (5) does the patient have limitation in ≥ 1 ADL? If the answer to any of these five questions is ‘yes’, the patient belongs to category 2 or 3. If the answer to all five questions is ‘no’, the patient belongs to category 1 (Antonini et al. 2021).

Patients belonging to category 2 or 3 have to be assessed in the second section of MANAGE-PD to determine their eligibility for DAT. The exact algorithm of MANAGE-PD can be accessed online through the supplementary files (Antonini et al. 2021).

The development of the MANAGE-PD is comparable to the 5-2-1 criteria (Antonini et al. 2018, 2021). The international Delphi panel formed the basis of the MANAGE-PD. However, the published reports do not reflect in what way the elements of the MANAGE-PD tool were selected from the 15 clinical indicators for advanced PD and the 7 clinical indicators for eligibility for DAT. It is not clear why the MANAGE-PD tool contains more items than the 5-2-1 criteria, and therefore it remains unknown whether MANAGE-PD is a parsimonious model (Moes et al. 2022).

Validation of the MANAGE-PD was based on the assessment of 10 hypothetical patient vignettes by 400 general neurologists and 17 PD experts. The judgment of these neurologists was compared with the outcome of the MANAGE-PD tool. The experts’ individual ratings agreed well with the MANAGE-PD (unweighted kappa: 0.77), while general neurologists’ ratings agreed moderately (unweighted kappa: 0.57). It is not possible to comment on the sensitivity, specificity, prevalence and positive predictive value in this validation study. Moreover, the percentage of DAT-eligible patients was high in the vignette-based study (50%) (Moes et al. 2022).

The same article also presents the results of the application of the MANAGE-PD in a leveraged cohort of 2546 patients. The description of the cohort is brief, although the supplementary Appendix D shows that 81.5% of the patients in this cohort were treated by a movement disorders specialist, 15.4% were treated by a general neurologist and 3.1% by an internal medicine doctor. As a result, the study population may not be representative for the practice of general neurologists. Furthermore, it is not possible to make a statement about the diagnostic accuracy of MANAGE-PD based on the data shown, because this study did not examine the extent to which the outcome of the tool corresponded to a reference test (gold standard), such as expert or treating physician opinion (Antonini et al. 2021).

The OBSERVE study by Fasano et al. (2022) used the clinical characteristics of the first section of MANAGE-PD as the definition for advanced PD (Delphi criteria for advanced PD). They examined 2615 consecutive patients in centers offering DAT in 18 different countries. Participating neurologists indicated whether they thought the patient had advanced PD (both their own judgment and the judgment according to the Delphi criteria [not independently assessed], and whether the patient was eligible for DAT. In total 2533 patients had sufficient data to assess whether they had advanced PD according to the stated Delphi criteria, resulting in 1968 patients (77.7%) with advanced PD. However, according to the opinion of the treating neurologist, 1293 patients (51%) had advanced PD (Fasano et al. 2022). This confirms data from a previous publication, which also showed a moderate correlation between the opinion of the treating physician on advanced PD and the classification based on indicators of the Delphi criteria (kappa: 0.44) (Fasano et al. 2019).

##### D-DATS

Currently, a new screening tool is developed in the Netherlands (Groningen), the so-called Dutch DAT Screening tool (D-DATS), supporting general neurologists to determine whether a PD patient is eligible for referral to hospitals offering a particular DAT (DBS, LCIG, or CSAI or LECIG) (Moes et al. 2023).

This study prospectively examined 259 consecutive PD patients who visited their neurologists for a follow-up visit at a general hospital (no centers of expertise). Clinical characteristics were recorded for all patients by both the physician and the patient using a questionnaire. The collected data were converted into anonymized patient vignettes. These vignettes were presented to a panel of 5 Parkinson experts. First, each expert evaluated the cases individually by assessing their eligibility for referral for DAT. The experts then met to discuss any vignettes that did not receive a unanimous “not eligible” vote. After discussion, the expert panel voted on each selected case, whereas the majority of votes determined the final decision.

The collected results were used to select predictors of “eligibility for referral for DAT” via logistic regression analysis. This eventually led to a multivariable regression model, which created the basis for the D- DATS tool. This screening tool resulted in a sum score of three factors, being levodopa equivalent daily dose (LEDD), presence of response fluctuations, and presence of troublesome dyskinesias.

The expert panel considered 17 patients out of 259 eligible for referral for DAT (point prevalence of 6.6%). The ROC curve of the D-DATS tool showed an area under the curve (AUC) of 0.97. At the chosen cut-off point, the sensitivity was 88%, specificity 94% and positive-predictive value 76%.

In the mean time, the D-DATS tool has been validated externally. The results of the validation study are expected mid-2023.

#### Screening tools for identifying DBS candidates

##### FLASQ-PD

To our knowledge, the FLASQ-PD (Florida Surgical Questionnaire for Parkinson Disease) has been the first tool developed to improve patient referral for DAT (Okun et al. 2004). The FLASQ-PD was designed as a triage tool for timely referral of PD patients eligible for DBS. The instrument consists of 5 sections with various questions about patient characteristics. It assesses whether the patient has idiopathic PD, whether there are contraindications or red flags and to what extent the patient characteristics make the patient a good candidate. These patient characteristics are rated on a scale of 0 to 34 points.

It is not clear how the FLASQ-PD was developed. The authors do not refer to a developmental study justifying the choice of questionnaire items, nor do they describe how the scoring system was created. Clinical accuracy and value for practice were tested through a validation study among patients referred for DBS (*N* = 174) (Okun et al. 2004). A total score of at least 25 points, and no red flags, fitted with the profile of PD patients being good candidates for DBS.

COMPRESS was a combined screening tool that used a weighted algorithm to calculate a score based on three pre-existing tools, namely (1) the FLASQ-PD, (2) a computer-based cognitive test called MindStreams^®^ and (3) several psychometric measures of mood, including the Geriatric Depression Scale (GDS) and the Zung Anxiety Self-Assessment Scale (ZASAS) (Oyama et al. 2012). COMPRESS indicated whether a PD patient was a good candidate for DBS. In summary, a pilot study of 19 patients showed that COMPRESS was reasonably consistent with the opinion of a movement disorder specialist (80%) and better than the impression of general neurologists. However, COMPRESS did not perform better than the FLASQ-PD, while completing the FLASQ-PD was less labor intensive.

In 2020, this was followed by publication of a Chinese translation and validation of the FLASQ-PD in a single-center retrospective study (Wang et al. 2020). The Chinese translation was the same in terms of content and number of sections, but the total score was 42 instead of 34 because a section with contraindications was added to the total score (absence of a contraindication counted as an extra point). The publication only partially discussed the diagnostic accuracy of the Chinese version of the FLASQ-PD. A total score of ≥ 28 points would result in 94.9% sensitivity identifying a patient who is a good candidate for DBS. However, the corresponding specificity for this cutoff point was not reported (Wang et al. 2020).

##### Stimulus 1

The Stimulus tool was published in 2009 (Moro et al. 2009). Stimulus is a two-part online decision-support tool developed using the RAND/UCLA Appropriateness method (a modified Delphi method). An international panel of 12 experts assessed 1728 theoretical, unique patient profiles, if they would be appropriate to be referred for DBS. The patient profiles were based on 9 relevant variables for DBS, based on the literature. The experts assessed appropriateness for referral using a 9-point scale, where appropriateness for DBS was defined as “the benefits of referral far outweighed the possible disadvantages”. Other aspects like costs and waiting lists had to be left out of the assessment. In the second stage of development, the number of possible variables was reduced to 7, which were assessed in 972 theoretical PD patient profiles.

Potential DBS candidates had to meet five absolute criteria in Part 1: (1) a diagnosis of idiopathic PD; (2) bothersome motor symptoms despite optimal pharmacological treatment; (3) marked motor improvement with l-dopa; (4) the absence of major medical conditions preventing surgery; and (5) the absence of major medically resistant mental disorders, such as depression or dementia. If these criteria were not met, the patients did not meet the requirements to consider DBS and no score was given. If a potential DBS candidate met all five absolute criteria in Part 1 of Stimulus, they were scored in Part 2 for seven key variables: age; duration of illness; severity of symptoms during the OFF-medication period; severity of dyskinesias; l-dopa-insensitive axial symptoms; refractory tremor; and intellectual disability. Once these were entered, the Stimulus program displayed a score from 1 to 9. Scores of 7 or higher were considered to be “appropriate for referral for DBS”. Scores of 4 to 6 were considered as “uncertain” and scores of 3 or less as “inappropriate for referral”.

Limitations of Stimulus are the lack of clarity on how the sum score is established when assessing the 7 variables. In addition, model development used categorized data from continuous variables, such as age. This makes the tool potentially less sensitive to certain age categories. Moreover, Stimulus was developed using 972 unique patient profiles. These patient profiles were generated based on the possible values of the 7 basic variables. This means that in the study group the proportions are known, for example 50% patients with disease duration < 5 years and 50% with disease duration ≥ 5 years, but also 33% patients without or with mild tremor, 33% patients with moderate tremor and 33% patients with severe tremor. This theoretical population is not necessarily equal to the real population of PD patients (Moro et al. 2009).

The Stimulus tool was reviewed for its use in daily practice in Germany and Spain (Wächter et al. 2011). The study compared the use of Stimulus with “care as usual” without any screening tool. The patients screened by Stimulus were more likely to be eligible for eventual DBS treatment as compared to the non-Stimulus selected patients (77 vs. 48%).

This study had a selection bias, because only referred patients underwent the reference test. At the same time, Stimulus was developed based on the decision of the expert panel as to whether patients were eligible for referral, whereas the reference test in this study was acceptance for DBS (Wächter et al. 2011).

##### Stimulus 2

The Stimulus tool underwent an update (Moro et al. 2016). The new tool is available online at: 

www.earlystimulus.eu

Basically, this study repeated the previous study (2009) with a larger group of 82 experts (71 neurologists and 11 neurosurgeons) from 28 countries around the world. They assessed 1296 theoretical patient profiles, build on 8 clinical variables. In the 2009 publication, 7 clinical variables were used, and at that time the variables age and disease duration were grouped differently (age categories in 2009: < 60, 60–69, ≥ 70 years; age categories in 2016: < 60, 60–74, ≥ 75 years; disease duration in 2009: < 5, ≥ 5 years; disease duration in 2016: 4–7 years, ≥ 7 years). In addition, a new clinical variable non-motor side effects of anti-Parkinsonian medication (no-light vs. moderate-severe) had been added. These theoretical patient profiles were reviewed for appropriateness for referral.

In total 46% of the reviewed cases were considered as eligible for referral for DBS, 15% unsuitable, and 39% as uncertain. Although the authors indicated that validation of Stimulus 2 was in progress, no validation data of this tool has been published so far (Moro et al. 2016).

### Head-to-head comparison

In 2014 the FLASQ-PD and Stimulus 1 were compared in a retrospective analysis of 147 consecutive PD patients newly referred for DBS (Coleman et al. 2014). Stimulus showed a higher AUC on the ROC curve compared to the FLASQ-PD (0.81 vs. 0.63, respectively). The FLASQ-PD had positive predictive values (PPV) of 38.1 and 50%, depending on the cutoff point chosen (at cutoffs of ≥ 15 and ≥ 25, respectively). The PPVs of Stimulus were estimated to be 41.6 and 61.3% at a score of  ≥ 3 or ≥ 7 points on Stimulus part 2, respectively. The reference test was the final judgement for eligibility for DBS by a multidisciplinary team after extensive evaluation of the patients. It should be noted that both the FLASQ-PD and Stimulus were evaluated in a modified form. The scores were calculated for all patients, even if the patients were deemed ineligible for by DBS in Part 1 of Stimulus. Moreover, this study did not use the predetermined cutoff points of both tools.

Overall, this study showed a higher diagnostic accuracy of Stimulus 1 vs. the FLASQ-PD. However, this outcome was influenced by a selection bias, because the study population consisted of patients referred for DBS evaluation. Therefore, the prevalence of eligibility for DBS was high in this cohort. This might have lead to an overestimation of the PPV (Usher-Smith et al. 2016).

The study of Moes et al. compared the D-DATS tool with the 5-2-1 criteria (disjunction: at least one criterion present) (Moes et al. 2023). This comparison showed that D-DATS, with 6.6% of patients being eligible for referral for DAT, had a PPV of 76%, whereas the PPV of the 5-2-1 criteria was only 20%. However, we have to wait for the external validation of the D-DATS tool to draw definite conclusions.

**Table 1:** Overview of tools and criteria for assessing advanced Parkinson's disease or determining referral eligibility for a device-aided therapy.

Tool name and type	First author and year	DAT-type	Intended user	Description	Development	Validation	Funding	Related articles
**CDEPA questionnaire** Screening tool for advanced PD	Luquin et al. 2017	Screening tool for advanced PD Implicitly: clinical indicators for DAT eligibility (DBS, CSAI and LCIG) Phrase in introduction of article: “Therefore, it is of interest to know the patients’ clinical characteristics that can define advanced PD and that make these patients eligible for device-aided therapies” Luquin et al. (2017)	Not explicitly specified in the article, but based on the study design and validation study presumably general neurologists treating patients with PD	A matrix of diagnostic criteria for advanced PD, with different symptom categories on the vertical axis (general characteristics, disability, motor symptoms, non-motor symptoms and neuropsychiatric symptoms), and three grades of certainty on the horizontal axis (definite, probable and possible). The diagnosis of advanced PD requires one definite symptom or two probable symptoms. A combination of possible symptoms may count as probable. No scoring system	3-round Delphi study (CEPA) to reach consensus on the definition of advanced PD and implicitly on the clinical indicators that determine eligibility for DAT. Delphi group participants included 240 Spanish neurologists including experts in movement disorders Luquin et al. (2017)	Martinez-Martin et al. (2018): 173 consecutive DAT-naïve PD patients with disease duration of at least 2 years in 24 Spanish hospitals. Eligibility criteria for study participation were assessed by a neurologist (#1) Reference test (gold standard): judgment of a neurologist (#1) on the disease stage according to prespecified criteria as established by the study group. Level of expertise by #1 not specified Index test: CDEPA questionnaire assessed by a neurologist (#2) who was blinded for reference-test judgment by #1 Prevalence advanced PD in study population: 37.6% Diagnostic properties: SENS: 96.9%, SPEC: 57.4%, PPV 57.8%	Luquin et al. (2017): AbbVie Spain S.L.U Martinez-Martin et al. (2018)*:* no funding source specified Martínez-Castrillo et al. (2021): AbbVie	Martinez-Martin et al. (2018) Martínez-Castrillo et al. (2021)
**5-2-1 criteria** Screening tool for advanced PD	Antonini et al. 2018	Screening tool for advanced PD	General neurologists	≥ 5 doses of oral levodopa per day and/or ≥ 2 h of “off” time per day, and/or ≥ 1 h of troublesome dyskinesia per day	4-stage Delphi-panel approach including 17 leading movement disorder specialists from Europe. 15 clinical indicators for advanced PD were identified, and 7 clinical indicators for DAT-eligibility	Malaty et al. (2022): 4714 DAT-naive patients from 7 countries Participating neurologists (*n* = 563; level of expertise not reported) evaluated up to 12 consecutive PD patients for inclusion in the study Outcome measure: advanced PD Reference test (gold standard): assessment of advanced PD by the treating neurologist Index test: meeting at least one of the 5-2-1 screening criteria Prevalence of advanced PD in study population (reference test): 14.9% [our calculation based on presented data] Diagnostic properties: SENS 78.6%, SPEC 75.2%, PPV 35.7% [our calculation based on presented data]	Antonini et al. (2018): AbbVie Malaty et al. (2022): AbbVie Odin et al. (2015): AbbVie	Malaty et al. (2022) Odin et al. (2015)
**MANAGE-PD** Screening tool for eligibility for DAT-referral	Antonini et al. 2021	Any DAT	General neurologists and nurse specialists	Online decision support tool. Available at http://www.managepd.eu	Similar to the 5-2-1 criteria, i.e. based on a 4-stage Delphi-panel study (Antonini et al. 2018). No information available on item selection	Antonini et al. (2021): (1) Assessment of 10 hypothetical vignettes by 400 general neurologists and 17 PD experts - Prevalence of DAT-eligible patients was 50% among these 10 vignettes (2) Application of MANAGE-PD in cohort of 2546 patients, but no comparison with a gold standard	Antonini et al. (2021): AbbVie Fasano et al. (2022): AbbVie	Fasano et al. (2022)
**D-DATS** Screening tool for eligibility for DAT-referral	Moes et al. 2023	Eligibility for referral for any DAT	General neurologists	A three-factor screening tool based on LEDD, response fluctuations, and troublesome dyskinesias	Prospective observational study of 259 consecutive, DAT-naïve PD patients Outcome: apparent eligibility for referral for DAT based on consensus by a panel of 5 experts in the field of DAT Multivariable logistic regression modelling to develop a screening tool for eligibility for referral for DAT. Logistic regression analysis for item selection Potential predictors were patient and disease characteristics as observed by attending neurologists	External validation pending	Moes et al. (2023): Dutch Parkinson Patient’s Association	
**FLASQ-PD** Screening tool for identifying DBS candidates	Okun et al. 2004	DBS	General neurologists and other healthcare practitioners who may refer patients for DBS	Screening tool on paper *5 sections* [A] criteria for diagnosis PD [B] potential contra-indications [C] general patient characteristics [D] favorable/unfavorable characteristics [E] medication trial information *Scoring system* 0–34 points (higher = better surgical candidate) Score ≥ 25 best surgical candidates 0–8 red flags. Red flags suggest an diagnosis other than idiopathic PD	Unknown. Not specified in publication	174 patients referred for surgical screening. Single center 107 patient with PD analyzed 8 surgery-ready	Okun et al. (2004): Michael J. Fox Foundation The National Parkinson’s Foundation Eric and Jennifer Scott	Okun et al. (2007) Oyama et al. (2012) Coleman et al. (2014) Wang et al. (2020) [Chinese translation of FLASQ-PD]
**Stimulus 1** Screening tool for identifying DBS candidates	Moro et al. 2009	DBS	General (community) neurologists	Electronic decision tool *Part 1: necessary to meet all five absolute criteria:* - A diagnosis of idiopathic PD - Bothersome motor symptoms despite Optimal pharmacologic treatment - Marked motor improvement with l-dopa - No major medical conditions that prevent surgery - No major medically resistant mental disorders *Part 2: score for seven key variables:* - Age - Duration of illness - Severity of symptoms during the OFF-Medication period - Severity of dyskinesias - l-dopa-insensitive axial symptoms Refractory tremor - and intellectual disability From the input data, Stimulus calculates a score from 1 to 9. Scores of 7 or higher are considered “appropriate”. Scores of 4 to 6 are “uncertain” and scores of 3 or less are “inappropriate”	Appropriateness method (a modified Delphi method) 12 international experts assessed 1728 theoretical, unique patient profiles for appropriate referral for DBS Patient profiles based on 9 variables considered relevant for DBS assessment	(Wächter et al. 2011) Comparison of Stimulus with “care as usual” in which patients were referred without initial screening through Stimulus	Moro et al. (2009): Medtronic International (development study: unrestricted) Wächter et al. (2011): Medtronic International Sàrl	Wächter et al. (2011), Coleman et al. (2014)
**Stimulus 2** Screening tool for identifying DBS candidates	Moro et al. 2016	DBS	General (community) neurologists	See Stimulus 1 for basic structure. Slight alterations. Available at http://www.earlystimulus.eu	See Stimulus 1 82 experts from 28 countries	None reported	Moro et al. (2016): Medtronic	

### Other screening tools and testing methods

The review above is a selection of published instruments and tools supporting adequate referral for DATs. We will briefly discuss the non-reviewed tools and explain why we did not choose to review them:

- CAPSIT-PD (Defer et al. 1999)
  The CAPSIT-PD is a patient evaluation program for PD patients who are candidates for surgical interventions. This evaluation program was developed on behalf of movement disorder specialists to determine if PD patients are eligible for DBS. One of its core requirements was that patients had to be diagnosed with PD for at least 5 years. We have not included CAPSIT-PD in our overview because it was not developed to support general neurologists in selecting patients for DATs.
- Kinesia™ wearable device (Heldman et al. 2016)
  Heldman et al. examined the added value of the Kinesia™ motion sensor in 28 patients to determine eligibility for DAT. The patients with a Kinesia™ sensor (*n* = 11) were more often assessed as suitable for DAT than patients assessed by a neurologist only (*n* = 17). We did not include the Kinesia™ in our review because of the small study size and the need to purchase the product, which is a barrier for general implementation.
- LEDD 1000 and/or ≥ 5 levodopa intakes per day (Barer et al. 2022)
  Barer et al. presented a pragmatic definition of PD patients receiving intensive therapy, existing of either oral levodopa ≥ 5 times/day and/or ≥ 1000 mg LEDD (calculation according to Tomlinson et al. 2010). A similar proposal to use LEDD ≥ 1000 mg as cut-off was suggested earlier (Dahodwala et al. 2020). Interestingly, other authors suggested a limit of LEDD ≥ 1100 mg, based on their observation that this is approximately the average LEDD in patients treated with DBS in trials (Weir et al. 2018). We did not include the definition of Barer et al*.* in our review, because the total LEDD does not fully characterize the state of the patient. Some PD patients, for instance women with a low BMI, are more vulnerable to the effects of levodopa and may experience choreatic peak-dose dyskinesias, even when the total LEDD is low.
- MNCD tool (García et al. 2022)
  This is a new classification system for disease stages in Parkinson’s disease. The scale consists of 4 main axes (M: motor symptoms; N: non-motor symptoms; C: cognition; D: ADL dependence). The MNCD was created by 16 Spanish neurologists, being inspired by the TNM classification (oncology) and the NIHSS scale (vascular neurology). We did not include the MNCD tool because the MNCD tool was not specifically developed to screen patients for DATs.

## Discussion and conclusions

We have provided an overview of the available decision rules to refer patients adequately and timely for DATs. To the best of our knowledge, this is the first review article that covers more than two screening methods and discusses the most important tools in detail. However, this is not a systematic review. We merely described available screening tools, but did not systematically analyze the risk of bias. This is important, because multivariable prediction models are preferably developed in accordance with the TRIPOD guideline (TRIPOD is an acronym for Transparent Reporting of a multivariable prediction model for Individual Prognosis or Diagnosis) (Collins et al. 2015). One possible method to determine the risk of bias in prediction model studies is PROBAST (Prediction model Risk of Bias Assessment Tool) (Moons et al. 2019).

Several arguments emerge from the literature as to why application of a user-friendly and accurate decision rule could potentially lead to better care. Over-referral should be minimized and patients who are eligible for DATs should be recognized timely, without unintentionally biasing certain groups, e.g. based on gender.

Several decision support tools are available and some are still under development. However, some of the existing tools have exclusively been developed to optimize DBS referral (FLASQ-PD and Stimulus), while others focused on screening for advanced PD (CDEPA and 5-2-1 criteria). Eligibility for referral for any DAT is screened by MANAGE-PD and D-DATS. However, very few studies compared the different tools. Therefore, this review is not able to conclude on which tool is most appropriate for application in daily practice. Furthermore, appropriate tools should demonstrate their internal consistency by providing reproducible data in different patient cohorts.

Future research should examine the overlap between advanced PD and eligibility for referral for DATs. Not all advanced PD patients are eligible for DATs. OBSERVE-PD data suggest that both concepts are different, but that advanced PD is a predictor of eligibility for referral for DAT (Fasano et al. 2019).

It is also essential that screening tools are evaluated in real-world practice, which should focus on non-specialist practices of general neurologists. This is important because the prevalence of the target condition determines the positive predictive value of a test (Usher-Smith et al. 2016). A high positive predictive value is particularly important because it implies that the proportion of inappropriate referrals will remain low. This prevents waiting lists from becoming congested with inappropriate referrals.

However, care should be taken to ensure that a high positive predictive value does not compromise sensitivity. After all, high sensitivity remains important to avoid missing PD patients who are appropriate candidates for DAT. Clearly, the balance between these two diagnostic properties should be tailored to the health care system in which the screening tool will be used.

The available tools should also be considered by non-neurologists, to support global implementation. Globally, it is quite common for PD patients not to be treated by a neurologist. In the United States, more than half of all PD patients were seen by general practitioners only (Willis et al. 2011). PD patients not treated by neurologists have been shown to receive suboptimal quality of care (Cheng et al. 2007).

With the increasing prevalence of PD, the shortage of neurologists and budget constraints may lead to undertreatment of many PD patients. These developments make simple and adequate screening tools even more important to maintain an appropriate standard of care. However, because available referral criteria do not appear to be widely used (Cabrera et al. 2021), it is important to continue to educate health care professionals about the use of accurate screening tools and to inform them about improved versions of these tools. Equally, accessible and sound patient education may increase awareness of DAT and contribute to shared-decision making (Nijhuis et al. 2019).

If future developments would result in longer waiting lists, adequate referral criteria will be needed to prioritize these waiting lists. Similar developments are already known in transplantation care, which obviously faces limited resources (Benvenuto and Arcasoy 2021).

Another unmentioned aspect of timely recognition of eligibility for referral for DAT is that eligible patients are not always interested in referral. The first Stimulus tool was used to analyze the willingness of patients to be referred to a specialized DBS center (Moro et al. 2009; Dinkelbach et al. 2017). Of the 264 patients who had a score of ≥ 6 on the Stimulus tool, only a minority (43.2%) consented to be referred (Dinkelbach et al. 2017). This may change if less invasive and more user-friendly treatments will become available.

We have to be keen on new predictive factors in screening patients for DATs. For example, a Canadian study found that patients taking more different types of anti-parkinsonian medication, were more likely to be eligible for DBS (Crispo et al. 2020). A Romanian study reported that patients treated with DAT (100% LCIG in Romania) were younger, had a longer disease duration, were more hours off per day and had more dyskinesia per day. In addition, they used more doses per day on average and had a higher LEDD. The DAT group also used more frequently amantadine and entacapone (Szász et al. 2019).

Very likely genetic screening will help in the near future to select patients for DATs (Salles et al. 2021, 2022). This was illustrated by the finding that patients with PD due to a genetic defect are overrepresented (up to 29%) in a DBS cohort, whereas only 5–10% had a genetic defect in the general population of PD patients (Angeli et al. 2013).

In conclusion, there will be an increasing need for accurate, uniform and user-friendly criteria for referral for DATs, taking into consideration the growing number of PD patients and the lack of experienced PD professionals (Dorsey and Bloem 2018; Zaman et al. 2021).

## Data Availability

Data sharing not applicable to this article as no datasets were generated or analysed during the current study.

## Abbreviations

CDEPA: Cuestionario De Enfermedad de Parkinson Avanzada
CSAI: Continuous Subcutaneous Apomorphine Infusion
DAT: Device-Aided Therapy
D-DATS: Dutch DAT Screening tool
DBS: Deep Brain Stimulation
FLASQ-PD: Florida Surgical Questionnaire for Parkinson Disease
LCIG: Levodopa-Carbidopa Intestinal Gel Infusion
LEDD: Levodopa Equivalent Daily Dose
PD: Parkinson’s Disease
SENS: Sensitivity
SPEC: Specificity
PPV: Positive Predictive Value
